# Are human endogenous retroviruses pathogenic? An approach to testing the hypothesis

**DOI:** 10.1002/bies.201300049

**Published:** 2013-07-17

**Authors:** George R Young, Jonathan P Stoye, George Kassiotis

**Affiliations:** 1 Division of Virology, MRC National Institute for Medical ResearchLondon, UK; 2 Division of Immunoregulation, MRC National Institute for Medical ResearchLondon, UK

**Keywords:** autoimmunity, cancer, endogenous retrovirus, integrations, mutagenesis, pathogenesis

## Abstract

A number of observations have led researchers to postulate that, despite being replication-defective, human endogenous retroviruses (HERVs) may have retained the potential to cause or contribute to disease. However, mechanisms of HERV pathogenicity might differ substantially from those of modern infectious retroviruses or of the infectious precursors of HERVs. Therefore, novel pathways of HERV involvement in disease pathogenesis should be investigated. Recent technological advances in sequencing and bioinformatics are making this task increasingly feasible. The accumulating knowledge of HERV biology may also facilitate the definition and general acceptance of criteria that establish HERV pathogenicity. Here, we explore possible mechanisms whereby HERVs may cause disease and examine the evidence that either has been or should be obtained in order to decisively address the pathogenic potential of HERVs.

## Introduction

A surprising finding from the sequencing of the human and mouse genomes was the overall proportion, around 45%, of transposable elements 1,2. Over 90% of these are retroelements (REs) 1, that can be broadly divided into two groups according to the presence or absence of long terminal repeats (LTRs). LTR-bounded elements, endogenous retroviruses (ERVs) and LTR-retrotransposons, comprise around 8% of the human genome 1. Although, once in the germ-line, ERVs can re-infect the host and thus amplify their copies, all ERVs can eventually be traced back to distinct events of germ-line infection by exogenous retroviruses. Many ERVs have been present in the germ-line for a period exceeding ten million years. During this time they will have undergone significant mutational change and will no longer encode infectious virus 3. There are however examples where both ERV and exogenous retroviruses can be found in the same species. These include both the mouse mammary tumor virus (MMTV) and Jaagsiekte sheep retrovirus (JSRV) 4, and the recently endogenized Koala retrovirus (KoRV) 5.

Exogenous retroviruses in many host species and replication-competent ERVs in certain species exhibit well-established pathogenic potential, through either insertional mutagenesis or interference of their products with host physiological processes. Therefore, human endogenous retroviruses (HERVs) have attracted greater attention with respect to association with disease. However, the potential mechanisms of HERV pathogenicity might not be identical with those of exogenous viruses. For example, no HERV has been shown to date to produce infectious virus. Thus, insertional mutagenesis by HERVs is an unlikely contributor to host pathology. Nevertheless, our increasing understanding of HERV biology is uncovering novel interactions with the host that could lead to pathology. Here, we review the mechanisms by which HERVs might cause or contribute to disease, assess the current evidence linking HERVs to pathogenesis, and propose a set of criteria that should be considered in establishing HERV pathogenicity.

## The retroviral life cycle involves genomic integration

Retroviral particles contain dimers of the single-stranded positive-sense linear RNA genome, which carries the coding sequences: group-specific antigen (*gag*), protease (*pro*), polymerase (*pol*), and envelope (*env*). The order of these is vital to the correct level of gene expression, subsequent cleavage and product formation, and is hence completely conserved amongst known retroviruses 6. ‘Simple’ retroviruses, such as murine leukemia viruses (MLVs), code only these sequences, whereas ‘complex’ retroviruses encode one or more additional accessory genes.

**Figure 1:**
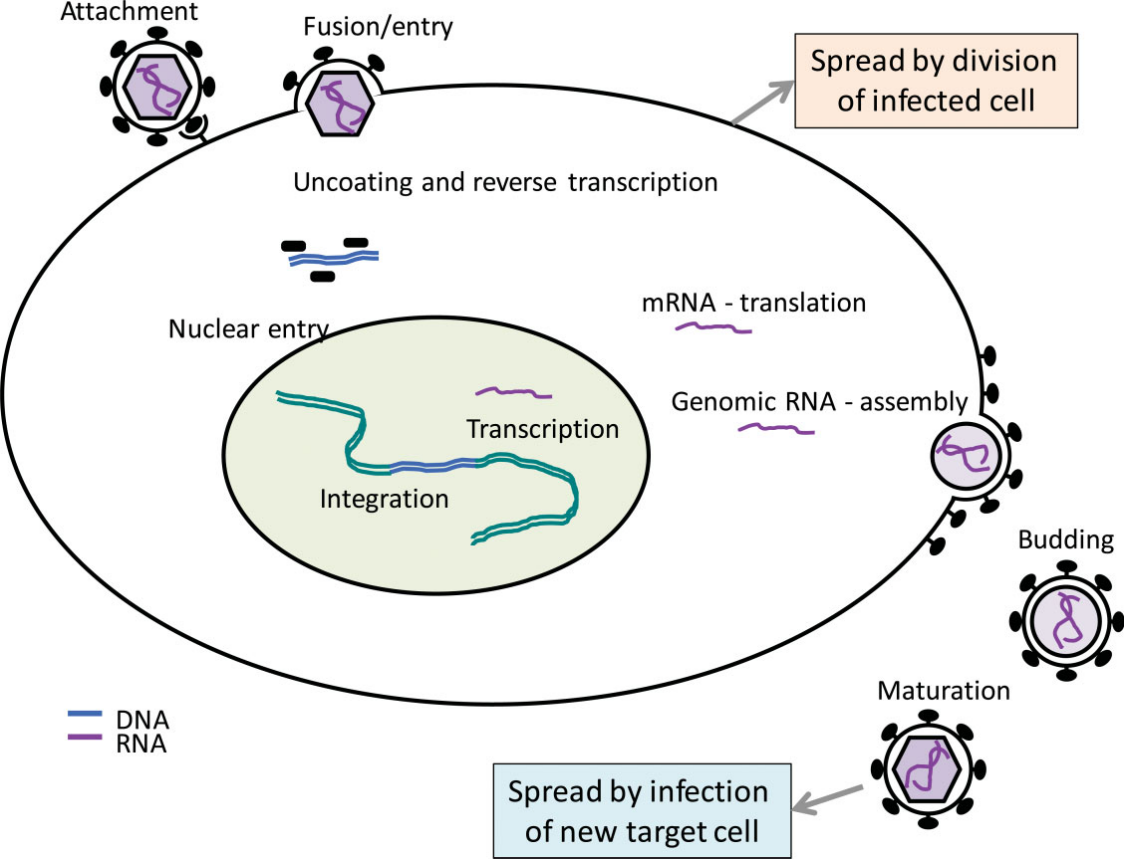
Typified retroviral life cycle. Retroviral infection begins with virion attachment usually to a cellular receptor, followed by fusion of virion and plasma membranes. In the cytosol, the two copies of genomic RNA are reverse-transcribed and following capsid disassembly they form the pre-integration complex, which then enters the nucleus. The reverse-transcribed cDNA copy is then integrated into the host cell DNA and from that point this provirus behaves analogously to a cellular gene, in that cellular division of an infected cell will create two infected daughter cells. Expression of mRNA from the provirus provides both new genomic RNAs as well as synthesis of viral proteins, which are all then assembled into new virions. These are then released from the plasma membrane and undergo maturation before they infect the next cell.

The defining feature of retroviral replication is the requirement for a proviral stage (Fig. 1). This feature necessitates two proteins – reverse transcriptase (RT) and integrase (IN), both encoded by the viral *pol* open reading frame. Virion binding to target cells leads to fusion of the viral and cellular membranes and release of the viral core into the cytoplasm. The viral RNA is subsequently reverse-transcribed with RT and prepared along with IN as the pre-integration complex for entry into the nucleus. For the majority of retroviruses, this process occurs only upon initiation of mitosis 7. Even for viruses that can infect non-dividing cells, this process is also substantially enhanced in actively-dividing cells. It is therefore expected that retroviruses have evolved mechanisms to induce activation and cell-cycle entry of the target cells and facilitate infection. One such example is superantigens encoded by MMTVs' *sag* gene, but other retroviruses may have evolved different mechanisms. This type of mitogenic effect of retroviruses may be a significant contributor to their pathogenicity.

**Figure 2:**
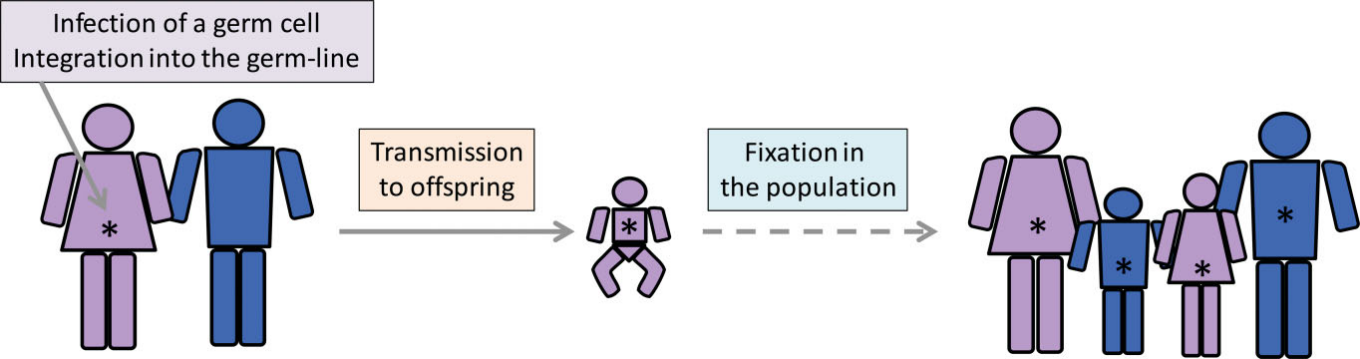
Model of retroviral endogenization. Retroviral infection of a germ cell is thought to make integration into the host germ line (denoted by an asterisk) possible. If an infected germ cell develops into offspring, it will transmit its provirus to every single cell of the offspring, akin to inheritance of a host gene. In evolutionary time, germ-line integrated proviruses can either expand in number within the germ-line and in the population, ultimately achieving fixation, or become extinct by random events or selection pressure against them.

Due to the requirement for a proviral stage in the retroviral life cycle, occasional infections in germ cells (Fig. 2) have preserved ERVs as a ‘fossil record’ of ancestral retroviral infections spanning many millions of years 8.

## Multiple HERV groups and members are distinguished

Although there is no standard nomenclature for HERVs, classification based on sequence homology to different genera of exogenous retroviruses has generally been adopted 9. HERV sequences broadly clustering with gamma and epsilon retroviruses have long been termed ‘Class I’, those clustering with beta retroviruses as ‘Class II’, and those having the greatest homology to spumaviruses as ‘Class III’. Naming of individual groups has generally centered on the predicted tRNA specificity of the binding site at which reverse transcription would initiate; a replication competent HERV-K virus would, e.g., use lysine. Around 30 such groups have been described based on a phylogenetic analysis of *pol* sequences; they vary in copy number from one to a few thousand 10, specific proviruses showing unique flanking sequences depending on their integration site.

**Figure 3:**
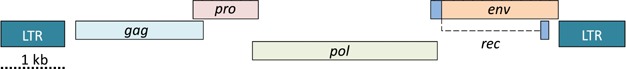
Exemplified HERV precursor genomic structure. This proviral structure is based on HERV-K(HML2) proviruses, which are considered the most recently introduced in humans (in the last five million years) and contain the most complete proviral copies, including full-length proviruses, with the most intact open reading frames (ORFs). The proviral LTRs can act as promoters of RNA transcription. Four major ORFs are depicted: *gag* encoding structural proteins; *pro* encoding protease; *pol* encoding RT, RNAse H, and IN domains; and *env* encoding for the retroviral envelope proteins. A smaller ORF, termed *rec*, is the functional counterpart of Rev and Rex encoded by more complex retroviruses, such as HIV-1 and HTLV-1, respectively. There appear to be several-hundred copies of proviruses belonging to this particular group in the human genome. However, in addition to very few copies of the near complete structure shown here, the majority harbor deletions ranging from small internal deletions to just solo LTRs, proviruses which have lost, by recombination between the two LTRs, all integral genes, leaving a single LTR sequence.

Contrary to species where subsets of ERVs bear similarity to currently infecting exogenous retroviruses, resemblance of HERVs to current human pathogens has not been demonstrated. The majority of HERVs are present in old-world monkeys, only the most recently integrated groups having some polymorphism between human populations and individuals 11. In particular, the HML2 subgroup of HERV-K (Fig. 3) contains proviruses that are found only in humans and also show some insertional polymorphism between individuals 12. It is evident that the different HERV groups, and individual members within, vary greatly with respect to genome structure and integrity and, consequently, their ability to produce RNA, protein, or even virions. However, each of these stages of the HERV life-cycle (Fig. 1), albeit incomplete, could in principle impact on a physiological process of the cell and although these are examined separately below, they could also act in a synergistic way.

## HERV DNA proviruses may affect genome function

HERV integrations may impact genome function even if not transcribed or translated 6. Depending on their position and transcriptional orientation in relation to a host gene, HERV LTRs can act as transcriptional promoters/enhancers or repressors of neighboring genes. HERVs that have integrated into introns can provide alternative transcription initiation or termination sites, resulting in truncated host gene mRNA transcripts. They can also provide additional splice donor or acceptor sites, either creating incorrectly spliced host mRNA or alter the ratio of physiological splice variants 13.

There is also accumulating evidence that HERVs might exert stronger influence on the expression of neighboring genes if they too are transcribed 14. In this case, the coding potential of the HERV RNA transcript is not relevant. Rather, disruption of the epigenetic silencing of a particular HERV provirus can activate or augment gene transcription. CpG DNA methylation is a central control of genome-wide transcriptional silencing and is established early in vertebrate development, but reversed in a specific and discrete fashion in distinct developmental pathways. This is thought to allow the expression of cell type-specific genes that confer identity and function. CpG DNA methylation also participates in the transcriptional silencing of HERVs and other REs, and it has long been known that CpG DNA demethylation can reverse the silencing of ERV LTRs 3.

Several types of cancer exhibit global DNA hypomethylation, which is considered critical in the activation of proto-oncogenes. Studies of methylation status in human cancers supported a model of widespread loss of epigenetic silencing also of REs, and in particular of more recently acquired groups 15. However, whether reversion of the transcriptionally silenced state of HERVs is causal to human cancers or simply a consequence of global DNA hypomethylation is more difficult to assess. An interesting insight has come from the study of Hodgkin's lymphoma, a B cell malignancy. Survival of malignant cells was found to require signaling from the colony-stimulating factor-1 receptor, encoded by the *CSF1R* gene 16. Although the *CSF1R* gene is expressed in a variety of hematopoietic cell-types, its transcription in Hodgkin's lymphoma cells was found to be initiated from an upstream LTR, which acted as an alternative promoter in this type of malignancy 16. This LTR belongs to the THE1B subgroup of mammalian apparent LTR retrotransposon (MaLR), and is normally silenced by DNA methylation. However, demethylation of this element was shown to cause its transcriptional derepression, leading to downstream *CSF1R* gene activation. Thus, altered expression or function of epigenetic modifiers together with availability of cellular transcription factors, such as the nuclear factor-κB (NF-κB), which can be attracted to the LTRs, may augment expression of proto-oncogenes.

In addition to influencing transcription of adjacent genes, HERV transcripts may also be part of the large and diverse group of regulatory RNAs. Several types of RNA can modulate gene expression through a variety of different mechanisms. These include microRNAs (miRNA) and small interfering RNAs (siRNA), both of which act through RNA interference, but can also affect target gene methylation. Long intergenic non-coding RNAs (lincRNAs) are non-protein coding transcripts thought to contribute to a complex regulatory network of gene expression. Accumulating evidence suggests that REs may be essential components of this system, providing an additional mechanism by which REs influence host gene transcription. A comprehensive study highlighted the intertwining nature of REs and human lincRNAs 17. Over 80% of lincRNAs were found to contain a RE, particularly enriched in HERVs, and more frequently inserted at the transcription start of the lincRNA 17. This finding suggests that HERVs are responsible for the transcriptional regulation of lincRNAs. Indeed, lincRNAs containing REs showed much more tissue-specific pattern of expression in comparison with lincRNAs devoid of REs 17. Of particular interest is the potential effect of lincRNAs containing HERV-H sequences, which are expressed specifically in stem cells 17. Non-coding RNAs transcribed from HERVs may also directly affect the activity of transcriptional repressors. The polypyrimidine tract-binding protein (PTB)-associated splicing factor (PSF) is a pre-mRNA splicing factor critically involved in the repression of several cellular genes, including proto-oncogenes. The repressive function of PSF can be inhibited by binding of non-coding RNA fragments, which ultimately leads in proto-oncogene activation, at least in cell lines. One of the non-coding RNA fragments that can bind PSF was shown to belong to a member of the HERVK11 group mapped as *MER11C* 18.

HERVs may additionally impact genome regulation and stability simply by providing regions of homology for DNA recombination. Chromosomal rearrangements have established causative roles in a variety of human conditions, including cancer, and can be caused by many different mechanisms. Non-allelic homologous recombination is a frequent cause of deletions and duplications, causing genomic disorders 19, and HERV homology can directly contribute to genomic rearrangements 20. Indeed, HERV15 elements have been found to provide the homology for the recombination event that removes the *azoospermia factor a* (AZFa) region of the Y chromosome, leading to male infertility 21,22. Also, loss of the *eyes absent 1* (*EYA1*) gene in branchio-oto-renal syndrome and recurrent unbalanced translocations leading to intellectual disability have been shown to result from recombination between HERV-H copies 23,24.

## HERV RNA transcription may trigger innate immunity

Infections with exogenous pathogens, including retroviruses, elicit innate immune responses, which are initiated by recognition of pathogen-associated molecular patterns by specialized receptors 25,26. Discrimination between pathogen and host products is based on the unique molecular signatures of pathogen products or their physical segregation in subcellular compartments 25,26. One such pathway relies on recognition of viral nucleic acids, either as viral genomes present in virions or viral genome replication intermediates generated in the infected cell. Receptors recognizing nucleic acids generated during various steps of the retroviral life-cycle have been well-characterized using exogenous retroviruses for infection 25. It stands to reason that, to avoid potentially damaging immune reactivity against the vast array of HERVs in our genomes, innate immune sensors would have evolved to recognize specific steps in the retroviral life-cycle that only replication-competent exogenous retroviruses can complete. Future work may uncover targeting by innate immunity of particular motifs or replication intermediates specific to replication-competent exogenous retroviruses.

Studies of human autoimmune or autoinflammatory conditions, including systemic lupus erythematosus (SLE) and the rare Aicardi-Goutières syndrome (AGS) have implicated a number of genes primarily involved in nucleic acid metabolism 27. These include the nucleases *3′ repair exonuclease 1* (*TREX1*), a DNA exonuclease that cleaves ssDNA fragments, and subunits of the *ribonuclease H2* (*RNASEH2*) trimer, an RNA nuclease that specifically degrades the RNA of RNA:DNA hybrids. They also include *adenosine deaminase*, *RNA-specific* (*ADAR*), which catalyzes the hydrolytic deamination of adenosine to inosine in double-stranded RNA (dsRNA), and *SAM domain and HD domain 1* (*SAMHD1*), an enzyme that hydrolyzes intracellular deoxynucleoside triphosphates (dNTPs). Importantly, all these enzymes have been demonstrated to affect distinct steps in HIV-1 replication, and it is speculated that they might play similar roles against HERVs. Indeed, analysis of *Trex1*-deficient mice has revealed accumulation of cytosolic DNA fragments derived from reverse-transcription of endogenous REs 28. These are then thought to trigger a cytosolic DNA sensor, leading to aberrant production of IFNs and, eventually, autoimmunity 29. Although the identity of human REs or HERVs whose replication intermediates might accumulate in cells deficient in TREX1, or indeed their potential to trigger pathogenic production of IFNs, has not been confirmed, it is speculated that similar accumulation of replication intermediates of endogenous REs in the absence of any of these nucleic acid metabolizing enzymes directly contributes to SLE and AGS development.

## HERV proteins can activate adaptive immunity

Despite the accumulation of replication-inactivating mutations, many HERVs still retain the potential to express retroviral proteins, which may affect cellular function and reactivity of both the innate and adaptive system. There is also evidence for a potential physiological role for some of these retroviral proteins, suggested by the retention in the human genome of more than a dozen *env* genes with full coding capacity 30–32.

Similarly to all other host proteins, expressed HERV proteins will inevitably provide antigenic epitopes for recognition by B or T cells. Mouse studies have revealed that ERV-encoded self-antigens can, in certain cases, mediate positive selection of developing thymocytes with specific T cell receptor (TCR) reactivity, and promote their peripheral response to cognate antigen 33,34. However, when the mechanisms that regulate TCR signaling threshold of thymocytes fail, the same ERV-encoded self-antigen induced an autoimmune response 33. More often, recognition of ERV-encoded self-antigens will induce a degree of immunological tolerance by deletion from the B cell receptor (BCR) or TCR repertoire of clones with sufficient reactivity to these antigenic self-epitopes. This would be expected to create ‘holes’ in the BCR or TCR repertoire, leaving the host unable to react to various exogenous antigens. Nevertheless, thymic selection by ERV-encoded self-antigens has also been shown to promote the avidity of the T cell response to exogenous antigens 35. The potential effects of ERV-mediated thymocyte selection, and consequently the ability of the host to respond to pathogens, is further complicated by the polymorphic nature of not only the ERVs, but also of the major histocompatibility complex (MHC) and TCR loci 35.

It is also clear, however, that endogenous retroviral antigens do not cause complete immunological tolerance and both T and B cell responses are frequently detected in both mice and humans 33,34,36–41. A significant amount of work has incriminated the envelope glycoprotein of certain endogenous MLVs as an autoantigen in murine SLE 42. HERV-encoded antigens have also been implicated as putative autoantigens in the development of human autoimmune diseases 36–39,43,44.

In addition to providing antigenic epitopes for lymphocyte recognition, certain HERV *env* sequences may also encode superantigens – proteins that stimulate T cells expressing specific TCR Vβ families. In particular, HERV-K18 *env* has been suggested to cause stimulation of Vβ7-expressing T cells in humans 45 and in transgenic mice 46. Owing to integration into the first intron of the *CD48* gene, encoding a lymphocyte-expressed member of the CD2 subfamily of immunoglobulin-like receptors, transcription of a copy of HERV-K18 can be induced by several stimuli that activate B cells, including EBV infection 47,48. Inducibility of HERV-K18 by viral infection also led to the suggestion that the superantigen activity encoded by particular alleles of this proviral integration is causality linked to the development of autoimmune diabetes 45. The proposed association, however, was not independently observed in several subsequent studies, casting doubt on the validity of the original observation 49.

## HERV proteins can be pathogenic

Exogenous present-day retroviruses encode an envelope protein that has been demonstrated to exert potent immunosuppression 50. A particular domain, termed immunosuppressive domain (ISD), has been identified within the transmembrane subunit of the envelope protein of Moloney MLV and Feline leukemia virus (FeLV), and was also shown to be relatively conserved among many different retroviruses of other species, including human 50,51. Immune suppression by the ISD appears necessary for efficient retroviral spread at least in mouse models 50. These findings support a potential role for HERV-encoded ISDs in immune modulation. Indeed, immunosuppressive potential has been demonstrated for the *env* of HERV-E 52, HERV-H 53, as well as *syncytins* 54. The latter genes are derived from HERV env genes, and their fusogenic properties are essential for placental development 54,55. Their immunosuppressive properties are also postulated to be involved in maternal immune tolerance of the fetus 54,55.

Evidence also exists to suggest that certain HERV envelope proteins can be immune-activating. Although elevated expression of several HERV *env* genes has been suggested for many neuroinflammatory diseases 56, HERV-W-encoded *syncytin* appears selectively upregulated in multiple sclerosis (MS) lesions 57. Expression of *syncytin*, as well as of HERV-H and HERV-K *env*, is upregulated during monocyte activation and differentiation, suggesting that it might represent a consequence of increased immune activation in these diseases, rather than a cause of neuroinflammation. However, syncytin protein expression in astrocytes induces production of proinflammatory cytokines and an increase in the oxidation of cellular proteins 57. This, in turn, leads to the damage and death of oligodendrocytes 57, a process considered central in MS pathogenesis, indicating a pathogenic contribution of HERV-W *syncytin*.

Emerging data suggest that in addition to nucleic acid and envelope protein, the retroviral capsid can also trigger innate immune responses and production of IFNs in particular. Incoming retroviruses are targeted by TRIM5α, which leads to capsid disassembly 58. In DCs, TRIM5α binding to the capsid lattice also triggers signal transduction cascades resulting in the activation of the transcription factors AP-1 and NF-κB 59. Although this is insufficient to induce IFN production alone, these cascades synergize with other pathways, thus enhancing the IFN response 59. TRIM5α shows high selectivity for retroviral capsids and the human isoform binds HIV-1 capsids only weakly 58. It has been proposed that the inability of human TRIM5α to restrict HIV-1 is the result of varying selection pressures amongst primates imposed by ancient retroviral infections, some of which gave rise to HERVs 60,61. It is, therefore, possible that human TRIM5α would bind HERV capsids. Indeed, human TRIM5α has been shown to bind the capsid of a resurrected ERV from the chimpanzee genome 61. However, this finding was not replicated in a separate study 62. Although not fully-infectious, HERV-produced virions may still enter cells, triggering TRIM5α-dependent signaling cascades. In addition to TRIM5α, Cyclophilin A (CypA), a cytoplasmic peptidylpropyl isomerase, has been shown to bind de novo synthesized capsids of HIV-1 and trigger an IFN response 63. However, CypA binding of capsid has only been seen with lentiviruses and the absence of a human endogenous lentivirus would suggest that this pathway is not relevant in the recognition of HERV capsids.

In addition to interacting with components of the immune system, HERV proteins can also interfere with other physiological systems and processes. For example, the Rec protein produced by the HERV-K(HML2) *rec* gene, an alternative splicing product of the HERV-K(HML2) *env* gene (Fig. 3), when expressed as a transgene in mice, interferes with germ cell development and causes lesions that resemble carcinoma 64. This effect of HERV-K(HML2) Rec is thought to result from association with the promyelocytic leukemia zinc-finger protein (PLZF) of the host, a transcriptional and chromatic regulator, and provides a paradigm of potentially tumor-promoting effects of other HERV proteins 12,65.

## Can HERVs produce potentially infectious virions?

The immune system is able to recognize and respond to products of nearly all steps of the retroviral life cycle, although there are certain immune pathways that are triggered more efficiently, if not exclusively, by virions and associated structures. For example, TLR7-mediated recognition of retroviral genomic RNA might be more efficient when virions reach the endosome, where nucleic acid-recognizing TLRs reside 66, and recognition of polymeric capsid proteins by TRIM5α is thought to occur shortly after virion entry and uncoating 58. Moreover, adaptive immune responses may also be stronger against the semi-crystalline protein structure of the virion than soluble proteins, and non-infectious virions are thought to represent the most potent antigenic form for the B cell response 67. It might, therefore, be reasoned that the pathogenic potential of an HERV that could produce even non-infectious virions is higher than that of an HERV that produces only unassembled proteins.

Evidence for virus-like particle production has been obtained in samples from many different autoimmune, inflammatory, hematopoietic, or neoplastic diseases 38, which has been used to argue for a causal effect. Although not every report of such virus-like particle production established the precise nature or origin of these putative virions, there are clear examples demonstrating HERV origin. Indeed, HERV-K(HML2) proviruses can produce virions in human germ-cell tumors 68–70, melanomas 71,72 or megakaryocytes cultured from essential thrombocythemia 73. However, the same proviruses are likely responsible for retrovirus-like virions found or induced in healthy tissues or cells. The presence of virions in human placenta 74–76 or breast milk 77,78 has long been proposed, and retrovirus-like particles can also be induced in transformed B cells from healthy donors 79.

In addition to potentially triggering cascades of innate and adaptive immune responses, the most obvious pathogenic mechanism that can be envisaged for a HERV is the production of infectious virions. These could then cause de novo infection and associated pathology via mechanisms that are relatively well understood for exogenous human and murine retroviral infections. However, none of the HERVs that have been studied to date have demonstrated the potential to produce infectious virions. Furthermore, genome sequencing approaches that have revealed novel somatic long interspersed nuclear element (LINE-1) integrations, failed to find HERV integration that would be consistent with re-infection 80. It remains theoretically possible that some of the more recently acquired HERV groups have extremely rare but intact variants in some human populations. For example, the HML2 group of HERV-K proviruses comprises the most recently active HERVs 12,81. From its approximately 60 full-length members, 11 HERV-K (HML2) proviruses are known to be insertionally polymorphic within the population, i.e., specific integrations are found only in a proportion of individuals 12,81. Although, selection would likely act against such integrations, rendering their frequency increasingly low, it is possible that a replication-competent HERV-K (HML2) provirus exists. Advances in genome and transcriptome sequencing technologies will facilitate the discovery of additional polymorphic HERV-K (HML2) proviruses and will ultimately establish the existence or otherwise of a replication-competent HERV.

## Recombination can generate replication-competent ERVs

Replication-competent retroviruses could also arise from a series of recombination events between two or more replication-defective HERVs, which would restore their respective defects. Due to the random nature of recombination between co-packaged retroviral genomes and the degree of genetic defects acquired by HERVs, the frequency of such an event would be vanishingly low. However, recent studies in mice have demonstrated that this frequency is increased considerably in certain types of immunodeficiency 82,83. Indeed, congenital immunodeficiencies affecting antibody production invariably lead to the spontaneous establishment of replication-competent pathogenic MLVs as a result of recombination between at least three replication-compromised proviruses. Interestingly, reconstruction of a consensus HERV-K (HML2) provirus by assembly and alignment of all of the complete, but defective HERV-K (HML2) proviruses resulted in a replication-competent recombinant retrovirus 84,85. HERV-K (HML2) proviruses can also naturally recombine 12,86, and, furthermore, recombination between three defective HERV-K (HML2) proviruses has been shown, at least in vitro, to generate an infectious retrovirus 84. Increased transcriptional levels such as have been observed following HIV-1 infection might be expected to favor recombination 87. Indeed, recent studies have provided evidence of frequent recombination of HERV-K (HML2) proviruses and accumulation of synonymous mutations in individuals infected with HIV-1, suggesting purifying selection of HERV-K(HML2) mutants or recombinants 87. It might be worth searching for novel proviruses in such people.

## What criteria can establish HERV pathogenicity?

Due to their considerable copy number in the genome, causality or even contribution may be more difficult to establish for many HERVs than for single etiologic agents. Fulfillment of Koch's postulates is a generally accepted criterion for establishing the causal relationship between an infectious microbe and a particular disease. However, two observations are not reconcilable in this frame of reference: firstly, most HERVs exist in the genome of all individuals; they are thus not uniquely present in disease. If such HERVs are considered infective agents, Koch's postulates are violated. Secondly, with the possible exception of an as-yet-undiscovered replication-competent member, the inability of all HERVs to replicate would also violate the second of Koch's postulates. HERVs might be viewed as genetic parasites, rather than transmissible infectious microbes, and, as such, investigation of their potential pathogenicity might require a modification of the original Koch's postulates. The molecular Koch's postulates are a set of criteria formulated by the microbiologist Falkow 88 based on Koch's postulates, to establish the causation of or contribution to disease by specific genes or virulence factors found in pathogenic microbes. These postulates could apply to HERVs that are insertionally polymorphic between individuals and their association with specific disease. Establishing association will also be facilitated by current work on genomic structure and potential function of these insertionally polymorphic HERVs 12. Moreover, HERVs integrated in the same location may also differ in primary sequence between individuals, and as such represent allelic variants that could be used in genetic studies. Indeed, classical genetic analysis has indicated linkage disequilibrium between polymorphisms around the *HERV-Fc1* locus and MS 89. More recently, Ian Lipkin has taken a somewhat different approach to this problem while trying to link causality with the discovery of novel pathogens 90. We suggest that the analysis of the potential role of a particular HERV in disease should be considered in four phases (Table1).

**Table 1:** Four phases of HERV pathogenicity consideration

A. Discovery – detection of disease related expression of specific HERV(s)
B. Identification – definition of individual proviruses involved
C. Description – development of a specific model for HERV involvement
D. Validation – testing and attempts to prove/disprove model

The first phase would involve detection of HERV expression in a manner consistent with disease involvement. This might involve an all-or-none effect (i.e. expression only in the disease group) or a quantitative effect (disease group expresses more than controls) though the degree of the difference might perhaps affect the enthusiasm for pursuing a viral etiology for the disease. Whether or not the provirus is expressed in normal subjects or tissues will have an important bearing on any subsequent models for disease involvement. Every effort should be made to detect expression levels of both RNA and protein.

Proviruses encoding expressed viral genes can be identified by sequencing the expressed transcript, provided this is accomplished using a technique like single genome amplification 91 to prevent in vitro recombination. The absence of precise matches in human sequence databases would prompt an immediate search for novel, possibly polymorphic HERVs or viral recombination events, either of which might provide clues for replication competent viruses with significant implications for mechanisms of disease. Provirus identification will allow genetic studies to see whether important functional domains of the virus/encoded protein are conserved. For example, an early candidate for a syncytin-like role could be excluded by examination of a large number of human sequences 92.

The third phase of this analysis would comprise construction of a detailed, specific model for HERV involvement in disease. Such models are frequently missing in studies making the case for HERV involvement based simply on analogies with replicating exogenous retroviruses. One important property of such a model would be a clear statement of whether or not the proposed mechanism involved virus replication. Virus replication implies the generation of novel integration sites; any mechanism dependent on new proviral DNA must document such sites or risk the embarrassment of an eventual discovery of a non-human origin for such sequences 93. Even a modest increase in copy number 94, should provide multiple new sequences when examined by next generation sequencing. If there is no difference in HERV content between normal and disease populations it is hard to see how that provirus can be directly causal. In these cases one must consider whether provirus expression is triggered by an external source and plays an important role in the disease process or simply reflects coincidental transcriptional activation of the provirus in a pathogenic setting.

Lastly, these models might be tested directly. It may be possible to set up animal models by expressing HERV sequences under the appropriate conditions, mimicking exposure from an endogenous element. Alternatively, one might consider attempts to modulate disease in humans by blocking viral protein function with antibody 95 or, if a good case can be made for involvement of a replication competent virus, with antiretroviral drugs. Ultimately, elucidation of the precise molecular pathway by which HERVs might cause disease would be the most convincing validation though it should be remembered that it may be easier to eliminate HERVs as potential agents of human disease than to prove that they are. Based on current evidence, many HERVs that have been postulated to play a role in disease pathogenesis would fail the proposed criteria.

## Conclusions and outlook

A pathogenic role for HERVs has long been hypothesized, but has remained difficult to prove conclusively. This difficulty may be partly due to the immense complexity of HERV biology, such as their repetitive nature and overabundance in the genome. However, difficulty may also stem from an expectation that HERV pathogenicity should involve mechanisms that are shared with fully-infectious exogenous retroviruses. Replication-competent HERVs may exist as very rare alleles or polymorphic insertions in the population, or emerge in pathological conditions, and evolving sequencing methodologies will certainly facilitate their discovery. However, HERVs can cause or contribute to disease via a variety of other mechanisms not involving infection that warrant further investigation. Study of the complex involvement of HERVs in regulating host gene expression will undoubtedly provide novel insights into their biology and potential pathogenicity. Particularly informative will be the dissection of the direct effects of HERVs on the transcription of adjacent genes, as well as the indirect effects on gene regulation through modulation of regulatory RNA networks. Current and future research is also expected to uncover more players in the interaction between HERVs and innate immunity. The replication-defective nature of HERVs may allow for the accumulation of replication intermediates that would not normally be found in abundance, and triggering of yet-undiscovered innate sensors. With increasing understanding of HERV gene structure and polymorphism, new genetic analysis of disease susceptibility can be designed taking into account the often multi-copy nature of HERVs. Also, the biological activity of proteins produced by HERVs, their interaction partners and pathways and potential effect on physiology and pathology is an area of considerable interest. Finally, inducibility of HERVs by external factors, such as microbial stimulation, also provides a potential mechanistic link between the environment, including commensals and pathogens, and disease development that would be important to explore.

## Abbreviations

AGS: Aicardi-Goutières syndrome
BCR: B cell receptor
Env: envelope
ERV: endogenous retrovirus
Gag: group-specific antigen
HERV: human endogenous retrovirus
IN: integrase
ISD: immunosuppressive domain
LINE: long interspersed nuclear element
LTR: long terminal repeat
MLV: murine leukemia virus
MMTV: mouse mammary tumor virus
MS: multiple sclerosis
NF-κB: nuclear factor-κB
Pro: protease
RE: retroelement
RT: reverse transcriptase
SLE: systemic lupus erythematosus
TCR: T cell receptor
